# Differences in risk factors between all-cause and pulmonary embolism-related death in acute pulmonary embolism: insights from the COMMAND VTE registry-2

**DOI:** 10.1016/j.rpth.2025.102965

**Published:** 2025-07-03

**Authors:** Soichiro Kobayashi, Yoshito Ogihara, Yugo Yamashita, Takeshi Morimoto, Ryuki Chatani, Kazuhisa Kaneda, Yuji Nishimoto, Nobutaka Ikeda, Yohei Kobayashi, Satoshi Ikeda, Kitae Kim, Moriaki Inoko, Toru Takase, Shuhei Tsuji, Maki Oi, Takuma Takada, Kazunori Otsui, Jiro Sakamoto, Takeshi Inoue, Shunsuke Usami, Po-Min Chen, Kiyonori Togi, Norimichi Koitabashi, Seiichi Hiramori, Kosuke Doi, Hiroshi Mabuchi, Yoshiaki Tsuyuki, Koichiro Murata, Kensuke Takabayashi, Hisato Nakai, Daisuke Sueta, Wataru Shioyama, Tomohiro Dohke, Toru Sato, Ryusuke Nishikawa, Takeshi Kimura, Kaoru Dohi, COMMAND VTE Registry-2 Investigators

**Affiliations:** 1 Department of Cardiology and Nephrology, Mie University Graduate School of Medicine, Tsu, Japan; 2 Department of Cardiovascular Medicine, Graduate School of Medicine, Kyoto University, Kyoto, Japan; 3 Department of Clinical Epidemiology, Hyogo College of Medicine, Nishinomiya, Japan; 4 Department of Cardiovascular Medicine, Kurashiki Central Hospital, Kurashiki, Japan; 5 Department of Cardiology, Toyonaka Municipal Hospital, Osaka, Japan; 6 Division of Cardiovascular Medicine, Toho University Ohashi Medical Center, Tokyo, Japan; 7 Department of Cardiovascular Center, Osaka Red Cross Hospital, Osaka, Japan; 8 Department of Cardiovascular Medicine, Nagasaki University Graduate School of Biomedical Sciences, Nagasaki, Japan; 9 Department of Cardiovascular Medicine, Kobe City Medical Center General Hospital, Kobe, Japan; 10 Department of Cardiovascular Center, Medical Research Institute Kitano Hospital, Osaka, Japan; 11 Department of Cardiology, Kinki University Hospital, Osaka, Japan; 12 Department of Cardiology, Japanese Red Cross Wakayama Medical Center, Wakayama, Japan; 13 Department of Cardiology, Japanese Red Cross Otsu Hospital, Otsu, Japan; 14 Department of Cardiology, Tokyo Women’s Medical University, Tokyo, Japan; 15 Department of General Internal Medicine, Kobe University Hospital, Kobe, Japan; 16 Department of Cardiology, Tenri Hospital, Tenri, Japan; 17 Department of Cardiology, Shiga General Hospital, Moriyama, Japan; 18 Department of Cardiology, Kansai Electric Power Hospital, Osaka, Japan; 19 Department of Cardiology, Osaka Saiseikai Noe Hospital, Osaka, Japan; 20 Division of Cardiology, Nara Hospital, Kinki University Faculty of Medicine, Ikoma, Japan; 21 Department of Cardiovascular Medicine, Gunma University Graduate School of Medicine, Maebashi, Japan; 22 Department of Cardiology, Kokura Memorial Hospital, Kokura, Japan; 23 Department of Cardiology, National Hospital Organization Kyoto Medical Center, Kyoto, Japan; 24 Department of Cardiology, Koto Memorial Hospital, Higashiomi, Japan; 25 Division of Cardiology, Shimada General Medical Center, Shimada, Japan; 26 Department of Cardiology, Shizuoka City Shizuoka Hospital, Shizuoka, Japan; 27 Department of Cardiology, Hirakata Kohsai Hospital, Hirakata, Japan; 28 Department of Cardiovascular Medicine, Sugita Genpaku Memorial Obama Municipal Hospital, Obama, Japan; 29 Department of Cardiovascular Medicine, Graduate School of Medical Sciences, Kumamoto University, Kumamoto, Japan; 30 Department of Cardiovascular Medicine, Shiga University of Medical Science, Otsu, Japan; 31 Division of Cardiology, Kohka Public Hospital, Koka, Japan

**Keywords:** anticoagulants, factor Xa inhibitors, mortality, pulmonary embolism, risk assessment

## Abstract

**Background:**

Accurate risk prediction of early mortality, particularly pulmonary embolism (PE)-related death, in patients with acute PE has become more important for selecting optimal management strategies.

**Objectives:**

To evaluate the cumulative 30-day incidence of and risk factors for all-cause and PE-related death within 30 days.

**Methods:**

In the COMMAND VTE Registry-2, which enrolled symptomatic patients with venous thromboembolism at 31 centers in Japan, we analyzed 2035 patients with acute PE.

**Results:**

The cumulative 30-day incidence of all-cause and PE-related death was 6.4% and 3.4%, respectively. Independent risk factors for all-cause and PE-related death were age >80 years (hazard ratio [HR], 2.43; 95% CI, 1.45-4.08; *P* < .001), hypoxemia (HR, 3.36; 95% CI, 1.07-10.5; *P* = .04), tachycardia (HR, 3.78; 95% CI, 2.20-6.50; *P* < .001), hypotension (HR, 5.43; 95% CI, 3.17-9.29; *P* < .001), an abnormal leukocyte count (HR, 1.78; 95% CI, 1.08-2.93; *P* = .02), and the absence of proximal deep vein thrombosis (HR, 2.58; 95% CI, 1.51-4.39; *P* < .001). Active cancer (HR, 2.59; 95% CI, 1.75-3.82; *P* < .001) and male sex (HR, 1.56; 95% CI, 1.07-2.28; *P* = .02) were independent risk factors for all-cause death, but not PE-related death. Chronic heart or lung disease (HR, 1.72; 95% CI, 1.02-2.90; *P* = .04) and right ventricular dysfunction (HR, 2.61; 95% CI, 1.02-6.70; *P* = .046) were independent risk factors for PE-related death, but not all-cause death.

**Conclusion:**

We identified several independent risk factors for PE-related death within 30 days, which differed from those of all-cause death. Risk factors specifically for PE-related death may be useful in decision-making for optimal treatment strategies for acute PE.

## Essentials

• Early mortality prediction, notably PE-related, is key for optimal PE care.
• We evaluated 30-day cumulative incidence and risk factors for all-cause and PE-related death.
• The cumulative incidence was 6.4% for all-cause death and 3.4% for PE-related death.
• Independent risk factors for PE-related death were distinct from those of all-cause death.

## 1 Introduction

Acute pulmonary embolism (PE) remains a critical health challenge due to its high morbidity and mortality [[1], [2], [3]]. In recent years, the management of venous thromboembolism (VTE) in the acute phase has undergone considerable changes, including a decrease in the use of thrombolytic therapy and inferior vena cava filters and an increase in the use of direct oral anticoagulants (DOACs) leading to outpatient treatment for some patients [4,5]. Furthermore, there is growing interest in the use of invasive endovascular treatments, such as catheter-directed mechanical thrombectomy for acute PE. Therefore, accurate risk prediction of early mortality in patients with acute PE has become more important in selecting optimal management strategies. There are currently several parameters and risk scores for predicting early mortality, including the Pulmonary Embolism Severity Index (PESI) [6], which are based on early all-cause death. In addition, more recently proposed indicators—such as the Prognostic Nutritional Index [7], the Naples Prognostic Score [8], and the pulmonary artery diameter to aorta diameter ratio [9]—have been suggested as potential tools for risk stratification. Nevertheless, the risk assessment of PE-related death may be more clinically relevant when tailoring optimal management strategies for acute PE. However, limited information is available on risk factors for PE-related death in the current era of DOACs; therefore, further studies are needed to obtain important insights for clinical decision-making regarding acute PE, as well as to establish a foundation for future research. To address this gap, the present study investigated risk factors for all-cause death as well as PE-related death 30 days after acute PE using a large-scale database in the DOAC era, which was collected before the introduction of catheter-directed mechanical thrombectomy.

## 2 Methods

### 2.1 Study design and population

The COMMAND VTE (Contemporary Management and Outcomes in Patients With VTE) Registry-2 is a physician-initiated, multicenter, retrospective cohort study. It enrolled consecutive patients with acute symptomatic VTE, confirmed objectively by imaging examination or autopsy, across 31 centers in Japan between January 2015 and August 2020. The design of the study was previously reported in detail [10]. Briefly, we enrolled consecutive patients who met the definition of acute symptomatic VTE diagnosed within 31 days of symptom onset during the study period [10]. The research protocol was approved by the relevant review boards or ethics committees at all 31 participating centers (Appendix 1). Informed consent was waived because we only used clinical information obtained during routine clinical practice. We provided information about the study on each hospital’s website and gave patients the opportunity to opt out. This study was conducted in accordance with the guidelines for epidemiological studies issued by the Ministry of Health, Labor, and Welfare in Japan.

**Figure 1:**
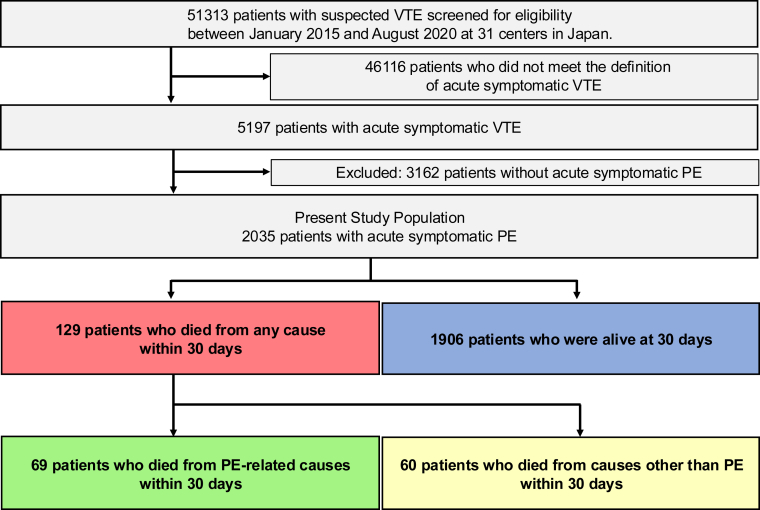
Study flowchart. Venous thromboembolism (VTE) included pulmonary embolism (PE) and/or deep vein thrombosis.

We enrolled 5197 consecutive patients with acute symptomatic VTE after screening 51,313 consecutive patients with suspected VTE based on chart reviews conducted by investigators at each institution. After excluding 3162 patients without acute symptomatic PE, the final study population consisted of 2035 patients with acute symptomatic PE who were categorized based on their survival status 30 days after the diagnosis of acute PE as follows: (1) those who died from causes directly attributable to PE; (2) those who died from causes other than PE; and (3) those who were alive (Figure 1). We analyzed patient characteristics and treatment strategies across the 3 types of patients. We also investigated risk factors for all-cause death as well as PE-related death 30 days after acute PE.

### 2.2 Data collection and definitions for patient characteristics

Baseline data were collected from hospital charts or databases according to prespecified definitions, utilizing an electronic case report form in a web-based database system. The study investigators at each institution were responsible for data entry, with automatic checks for missing or contradictory input and values outside the expected range. Additional editing checks were performed at the general office of the registry.

The severity of PE was classified as massive, submassive, or nonmassive, as previously proposed by the American Heart Association [11]. Shock was defined as systolic blood pressure < 90 mm Hg for at least 15 minutes, a pressure decrease of ≥ 40 mm Hg for at least 15 minutes, or the need for inotropic support. Hypoxemia was defined as an arterial oxygen partial pressure of < 60 mm Hg or an oxygen saturation of <90%. Tachycardia was defined as a pulse rate ≥ 110/min; if heart rate data were missing for patients with cardiac arrest or collapse, these patients were classified as having tachycardia. Hypotension was defined as systolic blood pressure < 100 mm Hg. If systolic blood pressure data were missing for patients with cardiac arrest, collapse, or shock, these patients were classified as having hypotension. Right ventricular (RV) dysfunction was defined as interventricular septum flattening and an estimated systolic pulmonary artery pressure > 40 mm Hg, as assessed by transthoracic echocardiography. Interventricular septum flattening was evaluated qualitatively in the short-axis parasternal view to establish whether it caused a D-shaped short-axis left ventricular (LV) cavity profile predominantly during systole. Additionally, RV dysfunction was defined as an obstruction index >50% and RV dilatation with an RV/LV ratio >0.90, as determined by contrast-enhanced computed tomography (CT) [12]. If RV dysfunction data were missing for patients presenting with cardiac arrest, collapse, or shock, these patients were classified as having RV dysfunction. Detailed definitions of other patient characteristics are provided in Supplementary Methods.

### 2.3 Clinical follow-up and endpoints

Follow-up information was gathered primarily through a review of hospital charts, supplemented by contacting patients, relatives, and/or referring physicians via phone and/or mail to inquire about their vital status, clinical events, invasive procedures, and status of anticoagulation therapy. An independent clinical event committee (Appendix 2), blinded to patient characteristics, reviewed all study outcomes. This committee classified causes of death as PE, bleeding events, cardiac causes, cancer, other causes, or unknown causes [13].

Death was classified as PE-related if confirmed by autopsy or if it followed clinically severe PE, either initially or after recurrent events. In more detail, PE-related death was classified based on the following 3 conditions: (1) autopsy-confirmed PE in the absence of another more likely cause of death; (2) death after objectively confirmed PE with imaging examinations in the absence of another more likely cause of death; (3) PE-related death not objectively confirmed, but highly suspected [14]. Death was classified as bleeding-related if it followed intracranial hemorrhage or a bleeding episode leading to hemodynamic deterioration. Death was classified as cardiac-related if it followed events such as myocardial infarction, heart failure, lethal arrhythmia, or sudden cardiac collapse. Death in patients with end-stage or advanced cancer without a specific cause of death was classified as cancer-related. Other deaths included those from causes such as respiratory failure or infection. Death without sufficient information to establish the cause was classified as death from an unknown cause.

### 2.4 Statistical analysis

Categorical variables were presented as numbers and percentages. Continuous variables were presented as means and SDs or as medians and IQR based on their distributions. The cumulative 30-day incidence of all-cause death was estimated using the Kaplan–Meier method. The cumulative 30-day incidence of PE-related death was estimated using the cumulative incidence function, considering death due to causes other than PE as a competing event. We constructed a multivariable Cox proportional hazard model to estimate the hazard ratios (HRs) and 95% CIs of potential risk factors for all-cause death at 30 days. Based on previous studies and clinical relevance, including each score component of simplified PESI (sPESI) [15], 13 potential variables at the diagnosis of PE were selected: age > 80 years, sex, chronic heart or lung disease, active cancer, hypoxemia, tachycardia, hypotension, the absence of proximal deep vein thrombosis (DVT), RV dysfunction, syncope, anemia, an abnormal leukocyte count (<4000 or >12,000), and D-dimer. Given the limited number of PE-related deaths, a stepwise selection method based on the same 13 potential variables was used to investigate potential risk factors for PE-related death at 30 days. A Fine–Gray subdistribution hazard model was applied to estimate HRs and 95% CIs for PE-related death at 30 days, considering death due to causes other than PE as a competing event. As a sensitivity analysis to explore the potential impact of treatment strategy, we reestimated HRs and 95% CIs for 30-day all-cause and PE-related death by including thrombolysis as an additional covariate. Additionally, in the subgroup of patients with submassive PE, we estimated the cumulative 30-day incidence of PE-related death according to the presence or absence of at least 1 of the independent risk factors for PE-related death identified in the present study, excluding RV dysfunction and hemodynamic instability, using the same cumulative incidence function. All statistical analyses were performed using EZR (Saitama Medical Center, Jichi Medical University), a graphical user interface for R (version 4.3.1, The R Foundation for Statistical Computing). All reported *P* values were 2-tailed, with significance set at a *P* value < .05.

## 3 Results

### 3.1 Patient characteristics

There were 129 patients (6.3%) who died from any cause within 30 days, 69 (3.4%) who died from PE-related causes within 30 days, and 1906 (93.6%) who were alive at 30 days (Figure 1). Among the 129 deaths, autopsy was performed in 2 patients, and autopsy imaging in 8 patients. In the entire study population, mean age was 67.3 years, and 41% were male (Table 1). Mean body weight and the mean body mass index were 60.9 kg and 24.0 kg/m^2^, respectively. Chronic heart or lung disease was present in 20% of patients, and 26% had active cancer, which was the most common comorbidity in patients who died from causes other than PE (73%) within 30 days, whereas it was less common in those who died from PE-related causes (26%) within 30 days.

**Table 1:** Baseline characteristics.

	All patients (*N* = 2035)	Patients who died from PE-related causes within 30 d (*n* = 69)	Patients who died from causes other than PE within 30 d (*n* = 60)	Patients who were alive at 30 d (*n* = 1906)
**Baseline demographics**				
Age, y	67.3 ± 15.2	67.9 ± 18.3	73.4 ± 10.1	67.1 ± 15.2
>80	408 (20)	22 (32)	15 (25)	371 (19)
Male	831 (41)	29 (42)	37 (62)	765 (40)
Body weight, kg	60.9 ± 15.2	60.9 ± 15.5	55.5 ± 12.2	61.1 ± 15.2
Body mass index, kg/m^2^	24.0 ± 4.9	23.4 ± 4.8	21.5 ± 3.7	24.1 ± 4.9
Body mass index ≥ 30 kg/m^2^	174 (8.6)	5 (7.2)	1 (1.7)	168 (8.8)
**Comorbidities**				
Active cancer	533 (26)	18 (26)	44 (73)	471 (25)
Hypertension	942 (46)	27 (39)	22 (37)	893 (47)
Diabetes	297 (15)	7 (10)	13 (22)	277 (15)
Dyslipidemia	542 (27)	9 (13)	9 (15)	524 (27)
Chronic kidney disease	388 (19)	15 (22)	13 (22)	360 (19)
Dialysis	14 (0.7)	1 (1.4)	1 (1.7)	12 (0.6)
Chronic heart disease	208 (10)	13 (19)	7 (12)	188 (9.9)
Heart failure	83 (4.1)	8 (12)	2 (3.3)	73 (3.8)
History of myocardial infarction	45 (2.2)	3 (4.3)	3 (5.0)	39 (2.0)
Atrial fibrillation	108 (5.3)	6 (8.7)	4 (6.7)	98 (5.1)
Chronic lung disease	234 (11)	11 (16)	7 (12)	216 (11)
COPD	60 (2.9)	5 (7.2)	2 (3.3)	53 (2.8)
Asthma	83 (4.1)	5 (7.2)	0 (0)	78 (4.1)
Chronic heart or lung disease	412 (20)	19 (28)	13 (22)	380 (20)
Autoimmune disorder	191 (9.4)	5 (7.2)	4 (6.7)	182 (9.5)
Antiphospholipid syndrome	33 (1.6)	0 (0)	0 (0)	33 (1.7)
Liver cirrhosis	12 (0.6)	1 (1.4)	1 (1.7)	10 (0.5)
Varicose veins	80 (3.9)	1 (1.4)	2 (3.3)	77 (4.0)
History of stroke	196 (9.6)	7 (10)	8 (13)	181 (9.5)
History of syncope	184 (9.0)	6 (8.7)	1 (1.7)	177 (9.3)
History of VTE	133 (6.5)	5 (7.2)	5 (8.3)	123 (6.5)
History of major bleeding	148 (7.3)	7 (10)	8 (13)	133 (7.0)
Transient risk factors for VTE	712 (35)	21 (30)	27 (45)	664 (35)
**Presentation**				
Hypoxemia	1207 (59)	66 (96)	43 (72)	1098 (58)
Tachycardia	490 (24)	54 (78)	21 (35)	415 (22)
Hypotension	372 (18)	53 (77)	14 (23)	305 (16)
Shock	283 (14)	55 (80)	6 (10)	222 (12)
Cardiac arrest/collapse	136 (6.7)	45 (65)	0 (0)	91 (4.8)
RV dysfunction by CT or TTE	1143 (57)	63 (91)	28 (47)	1052 (55)
Concomitant DVT	1499 (74)	26 (38)	38 (63)	1435 (75)
Proximal DVT in lower extremities	965 (47)	18 (26)	28 (47)	919 (48)
**Laboratory tests at diagnosis**				
Hemoglobin, g/dL	12.5 (10.7-14.0)	12.1 (10.5-13.6)	10.9 (8.9-12.4)	12.5 (10.9-14.0)
Anemia	937 (46)	36 (52)	45 (75)	856 (45)
Leukocytes, /μL	8470 (6600-10,900)	11,250 (8700-13,763)	11,010 (7800-15,625)	8300 (6500-10,700)
<4000 or >12,000	441 (22)	28 (41)	26 (43)	387 (20)
Platelet count, ×10^3^/μL	19.6 (15.6-25.0)	15.2 (10.7-18.6)	18.1 (13.5-24.0)	19.8 (15.8-25.2)
Thrombocytopenia	100 (4.9)	15 (22)	7 (12)	78 (4.1)
D-dimer, μg/mL	11.4 (5.9-23.0)	25.1 (12.5-42.2)	26.1 (12.1-37.3)	10.8 (5.7-21.7)
Hereditary thrombophilia	82 (4.0)	4 (5.8)	1 (1.7)	77 (4.0)
**Clinical severity classification of PE**				
Cardiac arrest/collapse	136/2035 (6.7)	45/69 (65)	0/60 (0)	91/1906 (4.8)
Massive	147/2035 (7.2)	10/69 (14)	6/60 (10)	131/1906 (6.9)
Submassive	860/2035 (42)	8/69 (12)	22/60 (37)	830/1906 (44)
Nonmassive	892/2035 (44)	6/69 (8.7)	32/60 (53)	854/1906 (45)
**sPESI**				
Score 0	303/2035 (15)	0/69 (0)	0/60 (0)	303/1906 (16)
Score ≥1	1732/2035 (85)	69/69 (100)	60/60 (100)	1603/1906 (84)
Score 1	626/2035 (31)	3/69 (4.3)	5/60 (8.3)	618/1906 (32)
Score 2	542/2035 (27)	7/69 (10)	26/60 (43)	509/1906 (27)
Score 3	415/2035 (20)	30/69 (43)	23/60 (38)	362/1906 (19)
Score 4	126/2035 (6.2)	19/69 (28)	4/60 (6.7)	103/1906 (5.4)
Score 5	17/2035 (0.8)	6/69 (8.7)	2/60 (3.3)	9/1906 (0.5)
Score 6	6/2035 (0.3)	4/69 (5.8)	0/60 (0)	2/1906 (0.1)

Categorical variables are presented as *n* (%). Continuous variables are presented as the mean ± SD or as the median (IQR) based on their distributions.

A history of major bleeding was diagnosed if the patient had a history of International Society of Thrombosis and Hemostasis major bleeding. Hypoxemia was defined as an arterial oxygen partial pressure < 60 mm Hg or percentage saturation of hemoglobin with oxygen <90%. Tachycardia was defined as a pulse rate ≥ 110/min. If heart rate data were missing for patients presenting with cardiac arrest or collapse, they were classified as having tachycardia. Hypotension was defined as systolic blood pressure < 100 mm Hg. If systolic blood pressure data were missing for patients presenting with cardiac arrest, collapse, or shock, they were classified as having hypotension. Shock was defined as systolic blood pressure < 90 mm Hg for at least 15 minutes, a pressure decrease of ≥ 40 mm Hg for at least 15 minutes, or the need for inotropic support. RV dysfunction was defined as interventricular septum flattening and estimated systolic pulmonary artery pressure > 40 mm Hg, as assessed by TTE. Interventricular septum flattening was evaluated qualitatively in the short-axis parasternal view to establish whether it caused a D-shaped short-axis left ventricular cavity profile predominantly during systole. Additionally, RV dysfunction was defined as an obstruction index >50% and RV dilatation with an RV/left ventricle ratio >0.90 by contrast-enhanced CT. If RV dysfunction data were missing for patients presenting with cardiac arrest, collapse, or shock, they were classified as having RV dysfunction. Anemia was defined as a hemoglobin level < 13 g/dL for males and < 12 g/dL for females. Thrombocytopenia was defined as a platelet count < 100 × 10^9^/L. Hereditary thrombophilia included protein C deficiency, protein S deficiency, and antithrombin III deficiency. Other detailed definitions of patient characteristics are described in the Supplementary Methods. The sPESI score included the variables of age older than 80 years, history of cancer, history of chronic cardiopulmonary disease, heart rate of ≥ 110 beats/min, systolic blood pressure < 100 mm Hg, and arterial oxygen saturation <90% at the time of diagnosis.

COPD, chronic obstructive pulmonary disease; CT, computed tomography; DVT, deep vein thrombosis; PE, pulmonary embolism; RV, right ventricular; sPESI, simplified pulmonary embolism severity index; TTE, transthoracic echocardiography; VTE, venous thromboembolism.

Regarding the severity of PE, 7.2% of the study population was classified as having massive PE, 42% as submassive PE, and 44% as nonmassive PE. The rate of nonmassive PE was lower in patients who died from PE-related causes than in those who died from causes other than PE and those who were alive at 30 days (8.7%, 53%, and 45%, respectively). Regarding sPESI scores, 15% of the population had a score of 0, whereas no patients who died from PE-related causes or from causes other than PE had a score of 0.

### 3.2 Treatment strategies

DOACs were administered to 1650 patients (81%) as oral anticoagulation therapy. A vena cava filter was inserted in 172 patients (8.5%), and 198 (9.7%) received thrombolysis therapy (Table 2). The percentage of patients requiring ventilator support and percutaneous cardiopulmonary support was higher in patients who died from PE-related causes than in those who died from causes other than PE and those who were alive at 30 days (51%, 5.0%, and 5.4%; 33%, 0%, and 2.8%, respectively).

**Table 2:** Treatment strategies.

	All patients (*N* = 2035) *n* (%)	Patients who died from PE-related causes (*n* = 69) *n* (%)	Patients who died from causes other than PE within 30 d (*n* = 60) *n* (%)	Patients who were alive at 30 d (*n* = 1906) *n* (%)
Initial parenteral therapy	1470 (72)	42 (61)	52 (87)	1376 (72)
Heparin	1447 (71)	40 (58)	52 (87)	1355 (71)
Single injection at diagnosis	95/1447 (6.6)	5/40 (13)	2/52 (3.8)	88/1355 (6.5)
Continuous injection	1352/1447 (93)	35/40 (88)	50/52 (96)	1267/1355 (94)
Fondaparinux	24 (1.2)	0 (0)	0 (0)	24 (1.3)
Thrombolysis	198 (9.7)	9 (13)	2 (3.3)	187 (9.8)
Inferior vena cava filter use	172 (8.5)	4 (5.8)	3 (5.0)	165 (8.7)
Ventilator support	140 (6.9)	35 (51)	3 (5.0)	102 (5.4)
Percutaneous cardiopulmonary support	76 (3.7)	23 (33)	0 (0)	53 (2.8)
Oral anticoagulation therapy	1902 (93)	10 (14)	32 (53)	1860 (98)
Vitamin K antagonist (warfarin)	252 (12)	4 (5.8)	3 (5.0)	245 (13)
DOAC	1650 (81)	6 (8.7)	29 (48)	1615 (85)
Dabigatran	3/1650 (0.2)	0/6 (0)	0/29 (0)	3/1615 (0.2)
Rivaroxaban	539/1650 (33)	2/6 (33)	7/29 (24)	530/1615 (33)
Initial intensive treatment: 30 mg/d	439/539 (81)	0/2 (0)	5/7 (71)	434/530 (82)
Maintenance dose: 15 mg/d	479/539 (89)	2/2 (100)	4/7 (57)	473/530 (89)
Maintenance dose: 10 mg/d	19/539 (3.5)	0/2 (0)	0/7 (0)	19/530 (3.6)
Apixaban	382/1650 (23)	0/6 (0)	2/29 (6.9)	380/1615 (24)
Initial intensive treatment: 20 mg/d	247/382 (65)	0/0 (0)	2/2 (100)	245/380 (64)
Maintenance dose: 10 mg/d	342/382 (90)	0/0 (0)	2/2 (100)	340/380 (89)
Maintenance dose: 5 mg/d	33/382 (8.6)	0/0 (0)	0/2 (0)	33/380 (8.7)
Edoxaban, mg/d	726/1650 (44)	4/6 (67)	20/29 (69)	702/1615 (43)
60	283/726 (39)	2/4 (50)	6/20 (30)	275/702 (39)
30	439/726 (60)	2/4 (50)	14/20 (70)	423/702 (60)
15	4/726 (0.6)	0/4 (0)	0/20 (0)	4/702 (0.6)
Concomitant medications at discharge				
Corticosteroids	230 (11)	2 (2.9)	6 (10)	222 (12)
Nonsteroidal anti-inflammatory drugs	138 (6.8)	2 (2.9)	4 (6.7)	132 (6.9)
Proton pump inhibitors/H_2_ blockers	1132 (56)	12 (17)	29 (48)	1091 (57)
Statins	367 (18)	2 (2.9)	4 (6.7)	361 (19)
Antiplatelet agents	128 (6.3)	7 (10)	3 (0)	118 (6.2)

Initial parenteral therapy included heparin (single or continuous injection), fondaparinux, and thrombolysis (urokinase or tissue plasminogen activator) within 10 days after diagnosis. Antiplatelet drugs included aspirin, ticlopidine, clopidogrel, prasugrel, ticagrelor, and cilostazol.

DOAC, direct oral anticoagulant; PE, pulmonary embolism.

### 3.3 Clinical outcomes

**Figure 2:**
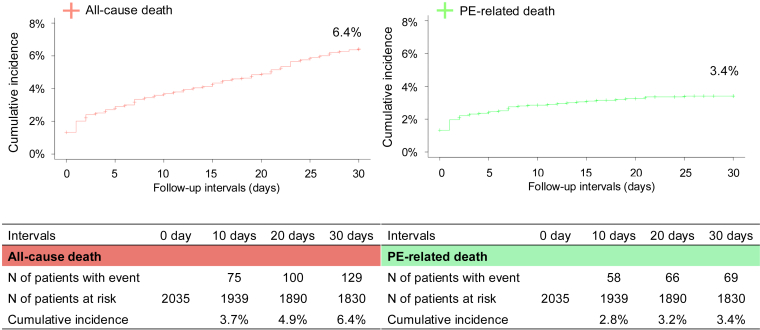
Cumulative incidence curves for all-cause death and pulmonary embolism (PE)-related death during the follow-up period of 30 days. The cumulative 30-day incidence of all-cause death was estimated using the Kaplan–Meier method. The cumulative 30-day incidence of PE-related death was estimated using the cumulative incidence function, with death due to causes other than PE treated as competing events.

The cumulative 30-day incidence of all-cause death and PE-related death was 6.4% and 3.4%, respectively (Figure 2). The most common cause of death within 30 days was PE (53%), followed by cancer (29%; Supplementary Table S1).

The multivariable analysis identified the following independent risk factors for all-cause death within 30 days: age >80 years (HR, 2.04; 95% CI, 1.35-3.07; *P* < .001), male sex (HR, 1.56; 95% CI, 1.07-2.28; *P* = .02), active cancer (HR, 2.59; 95% CI, 1.75-3.82; *P* < .001), hypoxemia (HR, 1.81; 95% CI, 1.05-3.10; *P* = .03), tachycardia (HR, 2.43; 95% CI, 1.60-3.67; *P* < .001), hypotension (HR, 3.46; 95% CI, 2.30-5.22; *P* < .001), an abnormal leukocyte count (HR, 1.75; 95% CI, 1.20-2.55; *P* = .004), and the absence of proximal DVT in the lower extremities (HR, 1.70; 95% CI, 1.16-2.50; *P* = .007; Table 3).

**Table 3:** Risk factors for all-cause death and pulmonary embolism-related death at 30 days.

Variables	All-cause death				PE-related death			
	Crude HR (95% CI)	*P* value	Adjusted HR (95% CI)	*P* value	Crude HR (95% CI)	*P* value	Adjusted HR (95% CI)	*P* value
Age >80 y	1.66 (1.14-2.43)	.009	2.04 (1.35-3.07)	<.001	1.90 (1.15-3.15)	.01	2.43 (1.45-4.08)	<.001
Male	1.53 (1.08-2.16)	.02	1.56 (1.07-2.28)	.02	1.05 (0.65-1.69)	.84		
Chronic heart or lung disease	1.31 (0.88-1.95)	.19	1.17 (0.75-1.81)	.48	1.50 (0.89-2.54)	.13	1.72 (1.02-2.90)	.04
Active cancer	2.64 (1.87-3.73)	<.001	2.59 (1.75-3.82)	<.001	0.99 (0.58-1.69)	.97		
Hypoxemia	3.90 (2.42-6.29)	<.001	1.81 (1.05-3.10)	.03	15.4 (4.86-48.9)	<.001	3.36 (1.07-10.5)	.04
Tachycardia	4.67 (3.28-6.63)	<.001	2.43 (1.60-3.67)	<.001	11.5 (6.53-20.3)	<.001	3.78 (2.20-6.50)	<.001
Hypotension	5.34 (3.77-7.55)	<.001	3.46 (2.30-5.22)	<.001	15.5 (8.92-26.9)	<.001	5.43 (3.17-9.29)	<.001
Absence of proximal DVT in lower extremities	1.67 (1.17-2.40)	.005	1.70 (1.16-2.50)	.007	2.60 (1.52-4.43)	<.001	2.58 (1.51-4.39)	<.001
RV dysfunction of CT or TTE	1.98 (1.34-2.92)	<.001	1.08 (0.68-1.73)	.73	9.49 (3.83-23.5)	<.001	2.61 (1.02-6.70)	.046
Syncope	0.58 (0.27-1.23)	.15	0.50 (0.23-1.09)	.08	0.96 (0.42-2.21)	.92		
Anemia	2.01 (1.40-2.87)	<.001	1.34 (0.90-1.99)	.16	1.28 (0.80-2.05)	.30		
Leukocytes (<4000 or >12,000)	2.72 (1.92-3.87)	<.001	1.75 (1.20-2.55)	.004	2.56 (1.58-4.13)	<.001	1.78 (1.08-2.93)	.02
D-dimer	1.01 (1.004-1.01)	<.001	1.00 (0.998-1.01)	.46	1.01 (1.004-1.01)	<.001		

Crude and adjusted HRs with their 95% CIs for all-cause and PE-related death at 30 days were estimated using a multivariable Cox proportional hazard model and a subdistribution hazard model, respectively. Based on previous studies and clinical relevance, we selected 13 variables as potential risk factors for all-cause death. Regarding PE-related death, a stepwise selection process was employed to refine the number of variables from the initial 13 variables due to the limited number of PE-related death events.

CT, computed tomography; DVT, deep vein thrombosis; HR, hazard ratio; PE, pulmonary embolism; RV, right ventricular; TTE, transthoracic echocardiography.

The multivariable analysis identified the following independent risk factors for PE-related death within 30 days: age >80 years (HR, 2.43; 95% CI, 1.45-4.08; *P* < .001), chronic heart or lung disease (HR, 1.72; 95% CI, 1.02-2.90; *P* = .04), hypoxemia (HR, 3.36; 95% CI, 1.07-10.5; *P* = .04), tachycardia (HR, 3.78; 95% CI, 2.20-6.50; *P* < .001), hypotension (HR, 5.43; 95% CI, 3.17-9.29; *P* < .001), RV dysfunction (HR, 2.61; 95% CI, 1.02-6.70; *P* = .046), an abnormal leukocyte count (HR, 1.78; 95% CI, 1.08-2.93; *P* = .02), and the absence of proximal DVT in the lower extremities (HR, 2.58; 95% CI, 1.51-4.39; *P* < .001; Table 3).

In the sensitivity analysis, including thrombolysis as a covariate, the set of independent predictors of all-cause death remained unchanged. As for PE-related death, chronic heart or lung disease was no longer retained in the model; however, thrombolysis was not identified as an independent predictor (Supplementary Table S2).

Among patients with submassive PE, the cumulative 30-day incidence of PE-related death was 1.1% in those with at least 1 identified risk factor and 0.0% in those without (Supplementary Figure).

## 4 Discussion

The main results obtained herein were as follows: (1) the cumulative 30-day incidence of all-cause and PE-related death was 6.4% and 3.4%, respectively; (2) several independent risk factors, such as age >80 years, hypoxemia, tachycardia, hypotension, an abnormal leukocyte count, and the absence of proximal DVT in the lower extremities, were common for both all-cause death as well as PE-related death within 30 days; (3) active cancer and male sex were independent risk factors for all-cause death but not PE-related death, whereas chronic heart or lung disease and RV dysfunction were independent risk factors for PE-related death but not all-cause death.

A previous study before the introduction of DOACs in Japan reported that the cumulative 30-day incidence of all-cause death among PE patients was 6.4% [16]. Another study, conducted between 1999 and 2018 in the United States, found that PE-related mortality rates remained unchanged at 3.4% to 3.5% despite advances in PE care [17]. Since event rates in the present study were similar to those in previous studies, acute mortality may not have changed significantly despite recent advances in PE treatment strategies. The appropriate selection of more aggressive treatment based on accurate predictions of acute PE-related death may be crucial for improving the prognosis of patients with acute PE.

The sPESI score, which has been widely used to predict all-cause death at 30 days, included established risk factors, such as age >80 years, hypoxemia, tachycardia, and hypotension [6,15]. The present study showed that these factors were correlated with both all-cause and PE-related death, reaffirming the relevance of the sPESI score for risk stratification in PE patients. An abnormal leukocyte count and the absence of proximal DVT in the lower extremities were also identified as significant risk factors for worse outcomes in the present study. Leukocyte counts have been associated with higher short-term mortality rates in various critical conditions, such as myocardial infarction, ischemic stroke, and sepsis [18,19]. Similarly, in the context of VTE, leukocytosis has been shown to increase the risk of mortality in cancer patients with VTE [20], in noncancer patients with VTE [21], and in patients with PE [22,23]. Additionally, a study on symptomatic patients with acute PE revealed that elevated and low leukocyte counts at admission were both associated with a higher risk of 30-day death [24]. These findings suggest the potential of an abnormal leukocyte count as a valuable prognostic risk factor in patients with acute PE. Furthermore, in patients with acute PE, the absence of proximal DVT may have reflected more severe hemodynamic compromise, potentially due to a larger clot burden embolizing the pulmonary arteries without leaving residual DVT in the lower extremities.

The present study highlighted distinct differences in risk factors for all-cause death and PE-related death. Active cancer and male sex were significant predictors of all-cause death but not PE-related death. Conversely, chronic heart or lung disease and RV dysfunction were significant predictors of PE-related death but not all-cause death. Active cancer is a well-established risk factor for overall mortality in patients with VTE [5,14,25]; however, its specific role in PE-specific mortality remains limited. A previous study identified active cancer as a predictor of both all-cause and PE-related death over a 3-month follow-up [25], which is in contrast to the present results, suggesting its prognostic impact for all-cause death only. This discrepancy may be attributed to differences in treatment strategies and patient populations, as well as follow-up durations. Early mortality in PE may predominantly be affected by very acute PE-specific factors, such as hemodynamic compromise, rather than malignancy itself, which may explain the limited relationship between cancer and PE-related death in our cohort. Sex differences in all-cause death have been reported, with some studies finding no significant differences between sexes [15,26,27], and others indicating higher short-term mortality in male sex [6,28], potentially due to a higher prevalence of comorbid conditions, such as atherosclerosis. Notably, these sex-related differences appear to be less pronounced for PE-specific outcomes, which may reflect the complex interplay between sex and comorbidity burden. Previous studies have demonstrated that chronic heart or lung disease was associated with all-cause death among PE patients [1,2,29]. However, this relationship was not observed in the present study, likely because milder forms of these conditions, such as bronchial asthma, were included. While chronic conditions such as chronic obstructive lung disease pose a significant risk due to a reduced pulmonary vascular reserve [30], the inclusion of milder cases may have weakened their overall impact on all-cause death. Previous studies consistently established RV dysfunction as a strong predictor of poor outcomes in PE patients, particularly for short-term mortality [[31], [32], [33]]. However, the present study showed that RV dysfunction was not associated with all-cause death, but correlated with PE-related death. A meta-analysis demonstrated that an RV/LV ratio ≥1.0 on CT was associated with a 2.5-fold increase in the risk of all-cause death and a 5-fold increase in the risk of PE-related death [34]. Furthermore, the 2019 European Society of Cardiology guidelines define RV dysfunction based on echocardiographic parameters, such as an RV/LV diameter ratio ≥1.0 or tricuspid annular plane systolic excursion < 16 mm, and CT-based parameters, such as an RV/LV ratio ≥1.0 [5]. In contrast, the present study applied a lower cutoff (RV/LV ratio >0.90 on contrast-enhanced CT), potentially including milder cases of RV dysfunction. These differences in the definition of RV dysfunction may partially account for the observed discrepancies in prognostic implications.

Importantly, 8.7% (6/69) of patients who died from PE within 30 days had initially been classified as having nonmassive PE. However, all 6 patients who died had a sPESI score of 1 or higher, and 5 of them had at least 1 of the clinical risk factors for PE-related death identified in the present study. These findings indicate that fatal PE may occur even in the absence of initial hemodynamic instability and RV dysfunction, underscoring the importance of early and comprehensive risk stratification. Additionally, among patients with submassive PE, the cumulative incidence of PE-related death was numerically higher in those with identified clinical risk factors than in those without. These findings suggest that patients with submassive PE who present with such risk factors—as well as those with massive PE—may be potential candidates for catheter-directed mechanical thrombectomy.

In the present study, we identified several independent risk factors for both all-cause and PE-related death within 30 days. As prognostic factors and scoring systems for PE become increasingly diverse [[6], [7], [8], [9],^15^], advanced analytical tools are needed to integrate complex clinical information. A recent study demonstrated that a deep learning-based model improved short-term mortality prediction in patients with acute PE compared with traditional risk scores [35]. Furthermore, applications of artificial intelligence in cardiovascular diseases, such as coronary artery disease and atrial fibrillation, has shown promise in enhancing diagnosis, risk stratification, and therapeutic decision-making [36]. Artificial intelligence-based approaches may thus provide opportunities to develop more refined, individualized risk prediction models for acute PE.

### 4.1 Study limitations

The present study has several limitations. As an observational study, treatment strategies, including the types and doses of anticoagulation therapy, were selected at the discretion of attending physicians, potentially affecting clinical outcomes. Furthermore, the present study was based on data from a Japanese cohort, which may limit generalizability due to potential differences in demographics, clinical practice, and clinical outcomes compared with those in other regions. In addition, the retrospective nature of the study may have led to the underreporting of clinical events, such as syncope and the absence of proximal DVT, particularly in fatal cases. Finally, in the present study, active cancer was not a significant predictor of PE-related death, but it is possible that deaths categorized as cancer-related may have included undetected PE-related contributions, especially in cases with progressive respiratory deterioration. However, these patients were in terminal stages of cancer, and the clinical course was consistent with cancer-related death.

## 5 Conclusions

We identified several independent risk factors for PE-related death within 30 days, which differed from those of all-cause death. Risk factors specifically for PE-related death may be useful in decision-making on optimal treatment strategies for acute PE.
