# Supplementary material for: Differences in risk factors between all-cause and pulmonary embolism-related death in acute pulmonary embolism: insights from the COMMAND VTE registry-2
Source: Res Pract Thromb Haemost. 2025 Jul 3;9(5):102965. doi: 10.1016/j.rpth.2025.102965 (PMC12336812; doi:10.1016/j.rpth.2025.102965)
Supplement: Supplementary Appendix [file mmc1.docx]

**e-Appendix 1: List of participating centers and investigators**

- Department of Cardiovascular Medicine, Graduate School of Medicine, Kyoto University, Kyoto, Japan: Kazuhisa Kaneda, Ryusuke Nishikawa, Yugo Yamashita (Kyoto University Hospital Ethics Committee; the approval number R3082)
- Department of Cardiovascular Medicine, Kurashiki Central Hospital, Kurashiki, Japan: Ryuki Chatani, Kazunori Mushiake, Kazushige Kadota (Kurashiki Central Hospital Ethics Committee; the approval number 3684)
- Department of Cardiology, Hyogo Prefectural Amagasaki General Medical Center, Amagasaki, Japan: Yuji Nishimoto, Yukihito Sato (Hyogo Prefectural Amagasaki General Medical Center Ethics Committee; the approval number 2021-3-50)
- Division of Cardiovascular Medicine, Toho University Ohashi Medical Center, Tokyo, Japan: Nobutaka Ikeda, Katsushi Amemiya, Masato Nakamura (Toho University Ohashi Medical Center Ethics Committee; the approval number H21053)
- Department of Cardiovascular Center, Osaka Red Cross Hospital, Osaka, Japan: Yohei Kobayashi, Ren Kimura, Tsukasa Inada (Osaka Red Cross Hospital Ethics Committee; the approval number J-0268)
- Department of Cardiovascular Medicine, Nagasaki University Graduate School of Biomedical Sciences, Nagasaki, Japan: Satoshi Ikeda, Yuki Ueno, Koji Maemura (Nagasaki University Ethics Committee; the approval number 21091305)
- Department of Cardiovascular Medicine, Kobe City Medical Center General Hospital, Kobe, Japan: Kitae Kim, Ryo Shigeno, Yutaka Furukawa (Kobe City Medical Center General Hospital Ethics Committee; the approval number 2021-09-17)
- Cardiovascular Center, The Tazuke Kofukai Medical Research Institute, Kitano Hospital, Osaka, Japan: Moriaki inoko, Shinya Ito (Kitano Hospital Ethics Committee; the approval number P211000100)
- Department of Cardiology, Kinki University Hospital, Osaka, Japan: Toru Takase, Gaku Nakazawa (Kinki University Ethics Committee; the approval number R03-170)
- Department of Cardiology, Japanese Red Cross Wakayama Medical Center, Wakayama, Japan: Shuhei Tsuji, Mamoru Toyofuku (Japanese Red Cross Wakayama Medical Center Ethics Committee; the approval number 2021-8-18)
- Department of Cardiology, Japanese Red Cross Otsu Hospital, Otsu, Japan: Maki Oi, Kazuaki Kaitani (Japanese Red Cross Otsu Hospital Ethics Committee; the approval number 650)
- Department of Cardiology, Tokyo Women's Medical University, Tokyo, Japan: Takuma Takada, Kentaro Jujo, Nobuhisa Hagiwara (Tokyo Women's Medical University Ethics Committee; the approval number 2021-0101)
- Department of General Internal Medicine, Kobe University Hospital, Kobe, Japan: Kazunori Otsui, Kenta Mori (Kobe University Hospital Ethics Committee; the approval number B210264)
- Department of Cardiology, Tenri Hospital, Tenri, Japan: Jiro Sakamoto, Toshihiro Tamura (Tenri Hospital Ethics Committee; the approval number 1228)
- Department of Cardiology and Nephrology, Mie University Graduate School of Medicine, Tsu, Japan: Yoshito Ogihara, Toru Sato, Kaoru Dohi (Mie University Hospital Ethics Committee; the approval number H2021-192)
- Department of Cardiology, Shiga General Hospital, Moriyama, Japan: Takeshi Inoue, Tetsuya Nadahama, Kunihiko Kosuga (Shiga General Hospital Ethics Committee; the approval number 20211119-02)
- Department of Cardiology, Kansai Electric Power Hospital, Osaka, Japan: Shunsuke Usami, Katsuhisa Ishii (Kansai Electric Power Hospital Ethics Committee; the approval number 21-067)
- Department of Cardiology, Osaka Saiseikai Noe Hospital, Osaka, Japan: Po-Min Chen, Toshiaki Izumi (Osaka Saiseikai Noe Hospital Ethics Committee; the approval number 20211103)
- Division of Cardiology, Nara Hospital, Kinki University Faculty of Medicine, Ikoma, Japan: Kiyonori Togi, Manabu Shirotani (Nara Hospital, Kinki University Faculty of Medicine Ethics Committee; the approval number 652)
- Department of Cardiology, Mitsubishi Kyoto Hospital, Kyoto, Japan: Kazuhisa Kaneda, Takafumi Yokomatsu (Mitsubishi Kyoto Hospital Ethics Committee; the approval number 21-28)
- Department of Cardiovascular Medicine, Gunma University Graduate School of Medicine, Maebashi, Japan: Norimichi Koitabashi, Hideki Ishii (Gunma University Hospital Ethics Committee; the approval number HS2021-138)
- Department of Cardiology, Kokura Memorial Hospital, Kokura, Japan: Seiichi Hiramori, Kenji Ando (Kokura Memorial Hospital Ethics Committee; the approval number 21082501)
- Department of Cardiology, National Hospital Organization Kyoto Medical Center, Kyoto, Japan: Kosuke Doi, Masaharu Akao (Kyoto Medical Center Ethics Committee; the approval number 21-034)
- Department of Cardiology, Koto Memorial Hospital, Higashiomi, Japan: Hiroshi Mabuchi (Koto Memorial Hospital Ethics Committee; the approval number 2021-05)
- Division of Cardiology, Shimada General Medical Center, Shimada, Japan: Yoshiaki Tsuyuki, Hiroto Yamamoto, Takeshi Aoyama (Shimada General Medical Center Ethics Committee; the approval number R3-11)
- Department of Cardiology, Shizuoka City Shizuoka Hospital, Shizuoka, Japan: Koichiro Murata, Eri Ishikawa, Ryuzo Nawada (Shizuoka City Shizuoka Hospital Ethics Committee; the approval number 2021-10-24)
- Department of Cardiology, Hirakata Kohsai Hospital, Hirakata, Japan: Kensuke Takabayashi, Mitsunori Miho, Shoji Kitaguchi, Takeshi Kimura (Hirakata Kohsai Hospital Ethics Committee; the approval number 2021-006)
- Department of Cardiovascular Medicine, Sugita Genpaku Memorial Obama Municipal Hospital, Obama, Japan: Hisato Nakai, Yuto Miura (Obama Municipal Hospital Ethics Committee; the approval number 2021-12-14)
- Department of Cardiovascular Medicine, Graduate School of Medical Sciences, Kumamoto University, Kumamoto, Japan: Daisuke Sueta, Kenichi Tsujita (Kumamoto University Hospital Ethics Committee; the approval number 2362)
- Department of Cardiovascular Medicine, Shiga University of Medical Science, Otsu, Japan: Wataru Shioyama, Yoshihisa Nakagawa (Shiga University Ethics Committee; the approval number R2021-094)
- Division of Cardiology, Kohka Public Hospital, Koka, Japan: Tomohiro Dohke (Kohka Public Hospital Ethics Committee; the approval number 2021-9-3)

**e-Appendix 2: Definitions for patient characteristics**

Patients with active cancer were defined as those undergoing cancer treatment, such as chemotherapy or radiotherapy, those scheduled for cancer-surgery, those with metastases to other organs, and/or those with terminal cancer (expected life expectancy of 6 months or less) at the time of the diagnosis [1]. Hypertension was diagnosed if peripheral blood pressure was >140/90 mmHg or if the patient was taking medication for hypertension. Diabetes mellitus was diagnosed using hemoglobin A1c (HbA1c) [National Glycohemoglobin Standardization Program (NGSP), 6.5%] as the standard or was assumed if the patient was taking medication for the treatment of diabetes. Dyslipidemia was diagnosed if total cholesterol was >240mg/dL, if high-density lipoprotein cholesterol was <40mg/dL, or if the patient was taking statins. Chronic kidney disease was diagnosed if there was persistent proteinuria or if estimated glomerular filtration rate (eGFR) was <60 mL/min/1.73 m^2^ for more than 3 months. The values of eGFR were calculated based on the equation reported by Japan Association of Chronic Kidney Disease Initiative [male 194*Scr^−1.094^*age^−0.287^, female: 194*Scr^−1.094^*age^−0.287^*0.739]. Dialysis included hemodialysis or peritoneal dialysis. Chronic heart disease was defined as persistent heart disorders including heart failure, history of myocardial infarction and atrial fibrillation. Heart failure was diagnosed if the patient had a history of hospitalization for heart failure, if the patient had symptoms due to heart failure [New York Heart Association (NYHA) functional class ≥2], or if the left ventricular (LV) ejection fraction was <40%. Chronic lung disease was defined as persistent lung disorders such as asthma, chronic obstructive pulmonary disease, and restrictive lung diseases. Chronic heart or lung disease was defined was persistent heart disorders including heart failure, a history of myocardial infarction, and atrial fibrillation or lung disorders such as asthma, chronic obstructive pulmonary disease, and a restrictive lung disease. An autoimmune disorder was defined as an immune-mediated disease, including inflammatory bowel disease, rheumatoid arthritis, or antiphospholipid syndrome [2]. History of major bleeding was diagnosed if the patient had a history of International Society of Thrombosis and Hemostasis (ISTH) major bleeding, which consisted of fatal bleeding, symptomatic bleeding in a critical area or organ, and bleeding causing a reduction in the hemoglobin level by at least 2 g/dL or leading to transfusion of at least 2 units of whole blood or red cells [3]. Transient provoking risk factors included major surgery with general anesthesia for greater than 30 min and within 2 months prior to the VTE, confinement to bed in the hospital with only bathroom privileges for at least 4 days with an acute illness within 2 months prior to the VTE, and a cesarean section within 2 months prior to the VTE, minor surgery with general anesthesia for less than 30 min and within 2 months prior to the VTE, admission to the hospital without confinement to bed due to an acute illness within 2 months prior to the VTE, estrogen therapy within 2 months prior to the VTE, pregnancy or puerperium within 2 months prior to the VTE, confinement to bed out of the hospital for at least 4 days within 2 months prior to the VTE, leg injury associated with reduced mobility for at least 4 days within 2 months prior to the VTE, long-distance travel lasting more than 6 h in the previous 3 weeks, central venous catheter use, and a coronavirus disease 2019 infection within 3 months prior to the VTE. Proximal deep vein thrombosis (DVT) in lower extremities was defined as venous thrombosis which was located in popliteal, femoral, or iliac veins. Anemia was defined as hemoglobin level <13 g/dL for male and <12 g/dL for female according to the standard World Health Organization classification of anemia [4]. Thrombocytopenia was defined as platelet count <100×10^9^/L [5]. Hereditary thrombophilia included protein C deficiency, protein S deficiency, and antithrombin III deficiency. The simplified PESI score included the variables of age greater than 80 years, history of cancer, history of chronic cardiopulmonary disease, heart rate of 110 beats/minute or greater, systolic blood pressure less than 100 mmHg, and arterial oxygen saturation less than 90% at the time of diagnosis. Initial parenteral therapy included heparin (single or continuous injection), fondaparinux, and thrombolysis (urokinase or tissue plasminogen activator) within 10 days after the diagnosis. Antiplatelet drugs included aspirin, ticlopidine, clopidogrel, prasugrel, ticagrelor, and cilostazol.

**e-Appendix 3: The independent clinical event committee**

- Yuji Nishimoto, MD: Hyogo Prefectural Amagasaki General Medical Center.
- Kosuke Doi, MD: National Hospital Organization Kyoto Medical Center.
- Kensuke Takabayashi, MD: Hirakata Kohsai Hospital.
- Ryusuke Nishikawa: Graduate School of Medicine, Kyoto University.
- Kazuhisa Kaneda: Graduate School of Medicine, Kyoto University.
- Yugo Yamashita: Graduate School of Medicine, Kyoto University.

**Supplementary Table 1. Causes of death within 30 days**

|  | **Causes of death**  **within 30 days**  **(N=129)** |
| --- | --- |
| PE related | 69 (53%) |
| Cancer | 38 (29%) |
| Cardiac events | 3 (2.3%) |
| Bleeding events | 2 (1.6%) |
| Other causes | 17 (13%) |
| Unknown causes | 0 (0%) |

Categorical variables were presented as number and percentages.

PE, pulmonary embolism.

**Supplementary Table 2. Risk factors for all-cause death and PE-related death at 30 days**

|  | **All-cause death** | | **PE-related death** | |
| --- | --- | --- | --- | --- |
| Variables | Adjusted HR  (95% CI) | P-value | Adjusted HR  (95% CI) | P-value |
| Age >80 years | 2.03 (1.35–3.06) | <0.001 | 2.37 (1.41–3.97) | 0.001 |
| Male | 1.57 (1.08–2.29) | 0.02 |  |  |
| Chronic heart or lung disease | 1.15 (0.74–1.78) | 0.54 | 1.64 (0.95–2.81) | 0.07 |
| Active cancer | 2.54 (1.72–3.75) | <0.001 |  |  |
| Hypoxemia | 1.85 (1.08–3.17) | 0.03 | 3.45 (1.11–10.8) | 0.03 |
| Tachycardia | 2.46 (1.62–3.72) | <0.001 | 3.83 (2.24–6.57) | <0.001 |
| Hypotension | 3.59 (2.38–5.42) | <0.001 | 5.68 (3.30–9.78) | <0.001 |
| Absence of proximal DVT  in lower extremities | 1.65 (1.12–2.43) | 0.01 | 2.52 (1.48–4.29) | <0.001 |
| RV dysfunction of CT or TTE | 1.11 (0.69–1.76) | 0.67 | 2.71 (1.05–6.96) | 0.04 |
| Syncope | 0.51 (0.24–1.11) | 0.09 |  |  |
| Anemia | 1.26 (0.84–1.90) | 0.27 |  |  |
| Leukocytes (<4,000 or >12,000) | 1.73 (1.19–2.53) | 0.004 | 1.72 (1.03–2.85) | 0.04 |
| D-dimer | 1.00 (0.998–1.01) | 0.42 |  |  |
| Thromboysis | 0.61 (0.32–1.17) | 0.13 | 0.60 (0.29–1.24) | 0.17 |

Adjusted HRs with their 95% CIs for all-cause and PE-related death at 30 days were estimated using a multivariable Cox proportional hazard model and a subdistribution hazard mode, respectively. Based on previous studies and clinical relevance, we selected 14 variables as potential risk factors for all-cause death. Regarding PE-related death, a stepwise selection process was employed to refine the number of variables from the initial 14 variables due to the limited number of PE-related death events.

CI, confidence interval; CT, computed tomography; DVT, deep vein thrombosis; HR, hazard ratio; PE, pulmonary embolism; RV, right ventricular; TTE, transthoracic echocardiograph.

**Supplementary Figure 1. Cumulative incidence curves for PE-related death in the sub-massive PE stratified by the presence or absence of at least one of the identified risk factors during the follow-up period of 30 days.**

The cumulative 30-day incidence of PE-related death was estimated using the cumulative incidence function, with death due to causes other than PE treated as competing events.

The identified risk factors consisted of age >80 years, chronic heart or lung disease, hypoxemia, the absence of proximal DVT, and an abnormal leukocyte count (<4,000 or >12,000).

DVT, deep vein thrombosis; PE, pulmonary embolism.

**Supplementary Reference**

[1] Sakamoto J, Yamashita Y, Morimoto T, Amano H, Takase T, Hiramori S, et al. Cancer-Associated Venous Thromboembolism in the Real World　- From the COMMAND VTE Registry. Circ J. 2019; 83: 2271-2281. <https://doi.org/10.1253/circj.CJ-19-0515>.

[2] Zoller B, Li X, Sundquist J, Sundquist K. Risk of pulmonary embolism in patients with autoimmune disorders: a nationwide follow-up study from Sweden. Lancet. 2012; 379: 244-249. <https://doi.org/10.1016/S0140-6736(11)61306-8>.

[3] Schulman S, Kearon C, Subcommittee on Control of Anticoagulation of the S, Standardization Committee of the International Society on T, Haemostasis. Definition of major bleeding in clinical investigations of antihemostatic medicinal products in non-surgical patients. J Thromb Haemost. 2005; 3: 692-694. <https://doi.org/10.1111/j.1538-7836.2005.01204.x>.

[4] . Nutritional anaemias. Report of a WHO scientific group. World Health Organ Tech Rep Ser. 1968; 405: 5-37.

[5] Yamashita Y, Morimoto T, Amano H, Takase T, Hiramori S, Kim K, et al. Influence of Baseline Platelet Count on Outcomes in Patients With Venous Thromboembolism (from the COMMAND VTE Registry). Am J Cardiol. 2018; 122: 2131-2141. <https://doi.org/10.1016/j.amjcard.2018.08.053>.
